# Novel Inducers of Fetal Globin Identified through High Throughput Screening (HTS) Are Active *In Vivo* in Anemic Baboons and Transgenic Mice

**DOI:** 10.1371/journal.pone.0144660

**Published:** 2015-12-29

**Authors:** Michael S. Boosalis, Jose I. Sangerman, Gary L. White, Roman F. Wolf, Ling Shen, Yan Dai, Emily White, Levi H. Makala, Biaoru Li, Betty S. Pace, Mehdi Nouraie, Douglas V. Faller, Susan P. Perrine

**Affiliations:** 1 Cancer Center and Hemoglobinopathy Thalassemia Research Unit, Boston University School of Medicine, Boston, Massachusetts, United States of America; 2 Department of Comparative Medicine, University of Oklahoma Health Sciences Center, Oklahoma City, Oklahoma, United States of America; 3 Phoenicia BioSciences, Weston, Massachusetts, United States of America; 4 Department of Pediatrics, Georgia Regents University, Augusta, Georgia, United States of America; 5 Department of Medicine, Center for Sickle Cell Disease, Howard University, Washington, DC, United States of America; Southern Illinois University School of Medicine, UNITED STATES

## Abstract

High-level fetal (γ) globin expression ameliorates clinical severity of the beta (β) hemoglobinopathies, and safe, orally-bioavailable γ-globin inducing agents would benefit many patients. We adapted a LCR-γ-globin promoter-GFP reporter assay to a high-throughput robotic system to evaluate five diverse chemical libraries for this activity. Multiple structurally- and functionally-diverse compounds were identified which activate the γ-globin gene promoter at nanomolar concentrations, including some therapeutics approved for other conditions. Three candidates with established safety profiles were further evaluated in erythroid progenitors, anemic baboons and transgenic mice, with significant induction of γ-globin expression observed *in vivo*. A lead candidate, Benserazide, emerged which demonstrated > 20-fold induction of γ-globin mRNA expression in anemic baboons and increased F-cell proportions by 3.5-fold in transgenic mice. Benserazide has been used chronically to inhibit amino acid decarboxylase to enhance plasma levels of L-dopa. These studies confirm the utility of high-throughput screening and identify previously unrecognized fetal globin inducing candidates which can be developed expediently for treatment of hemoglobinopathies.

## Introduction

The β-thalassemias and sickle cell disease (SCD), genetic disorders affecting the β-chain of adult hemoglobin A, are serious anemias and comprise a growing global health burden [1–24]. Fetal hemoglobin (HbF, α2γ2, *HBG*) is an endogenous hemoglobin present in all humans which is normally suppressed in infancy. Pharmacological augmentation of fetal hemoglobin (γ-globin) production, to replace the defective or missing β-globin chains, is a recognized therapeutic approach, as augmentation in HbF and F-cell levels reduce the severity of SCD, or reduce the ineffective erythropoiesis, and consequently the anemia, in β-thalassemia [2–23]. In SCD, HbF levels >15–20%, are required to ameliorate most of the clinical complications, as fetal globin (γ-globin) chains inhibit polymerization of sickle hemoglobin, preventing many pathologic consequences. While Hydroxyurea (HU) benefits many children and approximately half of adults[10–11] additional therapeutic agents would benefit those sickle cell patients who are Hydroxyurea intolerant or nonresponsive, and many thalassemia patients[6].

Pharmacologic reactivation of HbF expression offers a broadly applicable treatment approach for global diseases. Several classes of therapeutic agents induce fetal globin and HbF, including chemotherapeutic agents (Hydroxyurea (HU), 5-azacytidine, and Decitabine), short-chain fatty acids (SCFAs) and derivatives (SCFADs), histone deacetylase (HDAC) inhibitors, LSD-1 inhibitors, and factors that regulate globin translation[3–33]. Some of these therapeutics have clearly shown proof-of-principle, but, except for HU, have required parenteral administration, large doses, or require further clinical trials[2–33]. Non-cytotoxic agents which could be potentially used in combination with Hydroxyurea to produce additive efficacy, if necessary, would benefit many patients.

In studies here, a human cell-based assay utilizing a reporter gene (GFP), was adapted for high throughput screening (HTS) of chemical libraries to identify small molecules which induce fetal globin expression. Several libraries, including a library of therapeutic agents which are already FDA-or European Medicines Agency (EMA)-approved for other medical indications or in clinical trials currently were investigated for γ-globin-inducing activity. Candidate compounds which demonstrated initial γ-globin gene promoter activity were tested next for their ability to induce γ-globin mRNA expression and proportions of F-reticulocytes (cells expressing HbF protein) in human erythroid progenitors. A few candidate compounds found to have *in vitro* activity which also do not have undesirable side effects in their approved uses and are orally-bioavailable, were studied in anemic non-human primates, demonstrating 20-fold induction above pre-treatment values. The most active candidate was validated in transgenic beta YAC mice containing the human β-globin gene complex, with higher induction of F-cells (cells containing HbF protein) than was induced by Hydroxyurea (HU). Both animal models have been predictive of subsequent clinical activity for prior generation agents. These studies identified previously unrecognized, well tolerated, clinical-stage therapeutics which stimulate fetal globin expression and increase total hemoglobin levels in two animal models *in vivo*. As they are approved now for other medical conditions, Benserazide and Desloratidine offer an expedient route for clinical development in hemoglobinopathy populations.

## Methods

### High throughput screening (HTS) assay

**Fig 1:**
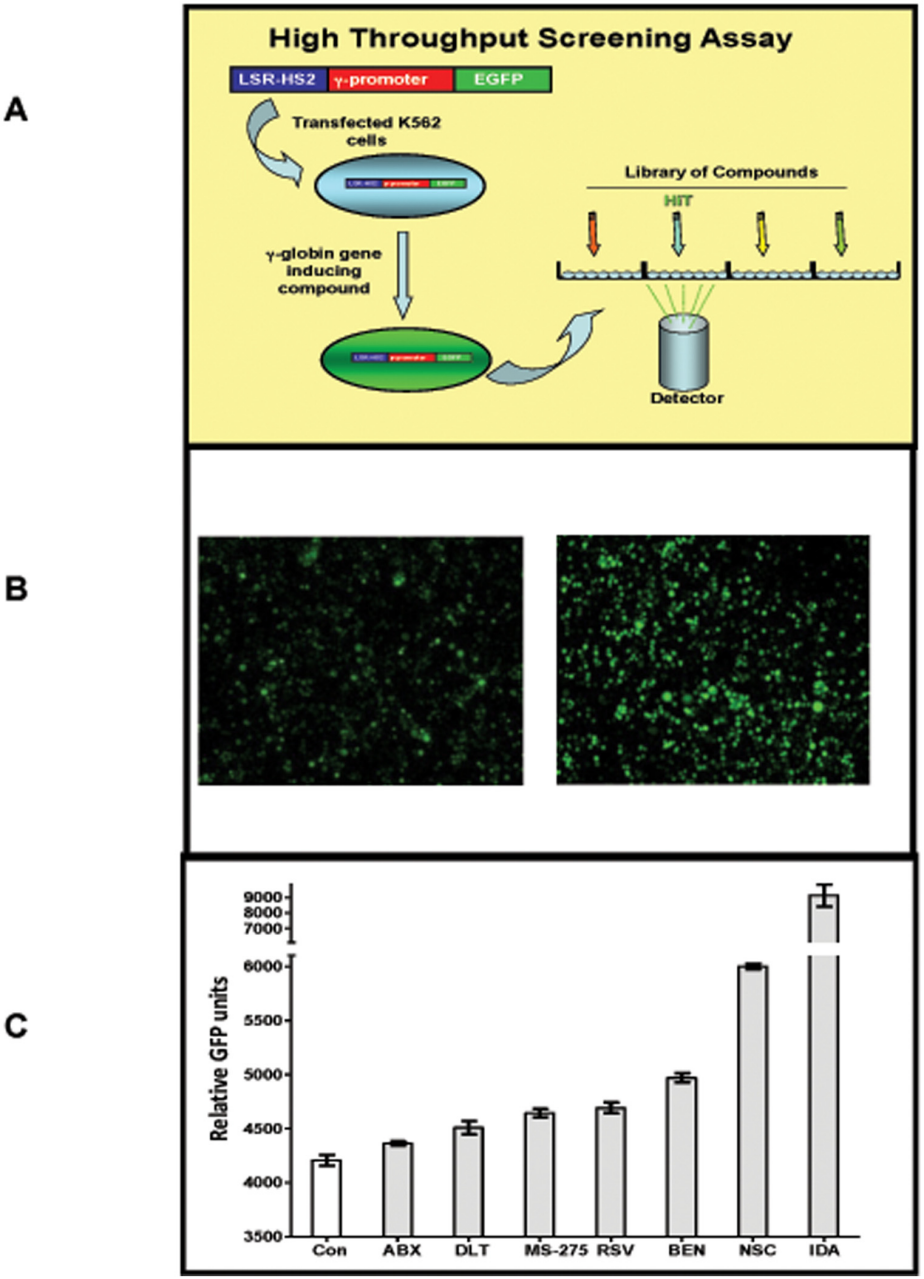
Schema of the high-throughput screening assay (HTS). (A.) A construct containing the 1.4-kilobase (kb) KpnI-BglII fragment of HS2 of the locus control region (LCR) linked to the γ-globin gene promoter driving the enhanced green fluorescent protein (EGFP) reporter gene stably transfected in K562 cells. Transfected cells were treated with compounds from diverse chemical libraries in an HTS format. (B.) Example of a hit in the high-throughput system is shown for compound MS-275. Fluorescent microphotographs of untreated cells are shown in the left panel; cells treated with MS-275 are shown in the right panel. (C.) Relative activity (relative fluorescence units) of new candidates identified by the HTS screen: Ambroxol (ABX), Desloratadine (DLT), MS-275, Resveratrol (RSV), Benserazide (BEN), NSC-95397 (NSC), and Idarubicin (IDA) are shown.

A high throughput screening (HTS) assay was developed using a cell-based reporter, stably transfected with a construct containing the 1.4-kilobase (kb) KpnI-BglII fragment of the human HS2 (hypersensitive sire 2) of the locus control region (LCR) linked to the γ-globin promoter and the enhanced green fluorescent protein (EGFP) reporter gene, as illustrated in Fig 1. Because EGFP messenger RNA (mRNA) is very stable, positive changes average 1.2- to 2-fold, and weak inducers are not detectable in this system [30]. Two-fold or higher induction over control indicates strong inducers of γ-globin gene activity[30]. The HTS assay was developed in 96-well format on a Tecan SpectraFluor Plus, incorporating multiple positive and negative control wells in each plate, generating 40–80 assay points for each. Optimization of the number of cells per well in a 96-well format was carried out and ideal time points for optimal fluorescence measurements were identified. In this 96-well format, a positive signal of intensity of 9000 RFUs was demonstrated in a volume of 100 μl. A signal-to-background ratio of at least 7 was demonstrated. The mean and standard deviations for the two controls were calculated, and the Z’ factor was generated (Z’ = 0.71) Two campaigns of 10,000 compounds were performed.

### Erythroid progenitor cultures and globin expression analyses

Erythroid progenitors were cultured from de-identified cord blood obtained from the New York Blood Center using CD34+ cells enriched using Ficoll-Paque PLUS (GE Healthcare, Piscataway, NJ) and EasySep (StemCell Technologies, Vancouver, BC), as previously described[28,30,31]. RNA was extracted and quantitative real time (qRT)-PCR was performed as previously described [28,30]. Briefly, cDNA was generated from equal amounts of total RNA extracted using the PerfectPure RNA purification kit (5 Prime Inc. Gaithersburg, MD) or RNA STAT-60 isolation reagent (Teltest, Friendswood, TX). Real-time PCR was performed using an ABI 7500 Real-Time PCR system (Applied Biosystems, Foster City, CA), with γ-globin mRNA calculated by the ΔΔCt method. The following primer set was used for γ-globin gene amplification: TCACAGAGGAGGACAAGGCTA and GAGATCATCCAGGTGCTTT. GAPDH and 18S mRNA levels were used for standardization.

For immunoblotting, erythroid progenitor cells on day 14 of Phase 2 culture were lysed in Laemmli sample buffer and subjected to 12% SDS-PAGE with constant voltage at 30 V for 3 hr. Proteins were transferred to a nitrocellulose membrane and probed with antibodies to HbF (sc-21756, Santa Cruz Biotechnology, Dallas, Texas) and β-actin (Sigma A, St. Louis, MO)[34]. Proteins were visualized with the GE Imaging System (Image Quant, LSD4010 (GE Healthcare, Piscataway, NJ) and quantified with ImageJ software (NIH, Bethesda, MD)[34].

### *In vivo* studies in anemic non-human primates

Studies to evaluate globin expression with treatment with the lead test candidates were performed with approval of the Institutional Animal Care and Use committee of the University of Oklahoma Health Science Center, employing juvenile baboons (*Papio hamadryas anubis*) from the breeding colony of the University of Oklahoma. Only baboons which adapt well to research environment are used. The baboons are maintained with indwelling vascular catheters, placed under general anesthesia, which are protected in a vest and swivel that allows free movement and in an environment with enrichment toys and companionship. Briefly, animals were chronically phlebotomized (by 3.7 to 5 mls/kg/day, to achieve and maintain stable anemia with total hemoglobin of 7.0 to 7.5 g/dl, as previously described [15]. This magnitude of phlebotomy reproduces stress erythropoiesis which occurs in the hemoglobinopathies, and exchanged the blood volume approximately every 10–20 days. Test drug candidates were administered based on the more rapid metabolism of the baboon compared to humans and projected human equivalent doses, based on previously used human doses as follows: Desloratadine was administered orally (0.5 mg/kg/dose), three times a week over two weeks. MS-275 (Selleckchem, Houston, TX) was administered orally three times a week for two weeks at doses of 0.2 mg/kg/dose. Benserazide (Enzon, Farmingdale, NY) was administered orally, at 1 mg/kg for 4 days/for one week or 2 mg/kg, 4 days per week, for two weeks. A washout period was provided between administration of different compounds in the same baboon, and each drug candidate was tested in at least 2 baboons. Results were compared to Hydroxyurea administered for 4 days/week at 25 mg/kg/dose (125 mg/day) over 3 weeks. Complete blood counts were performed 3 times per week. Assays of γ-globin mRNA, total hemoglobin, and % F-cells were assessed before and during treatment with test compounds. No animals were sacrificed for these studies.

### Analysis of F-cells by flow cytometry

Flow cytometry was performed on the baboons’ peripheral blood as previously described[15]. Briefly, cells were washed with PBS containing 0.1% BSA, fixed with 0.05% glutaraldehyde (Polysciences Inc Warrington, PA) for 10 minutes at room temperature, washed twice in 0.1% BSA in PBS, and cells were permeabilized with 0.1% Triton-X100 (American Bioanalytical, Natick, MA) for 3 minutes at room temperature. Cells were then washed, re-suspended in 0.1% BSA in PBS and dispensed in 5 x10^5^ cells/tube. PerCP isotype-labeled and unstained cells were used as controls. Thiazole orange was used to identify proportions and populations of reticulocytes. A custom-synthesized PerCP mouse anti-human antibody (BD Biosciences, San Jose, CA) that detects HbF-containing cells in the baboon was used to label fetal globin-containing cells. Samples were incubated in the dark at room temperature for 30 minutes, washed several times with 0.1% BSA in PBS, and analyzed by flow cytometry, using a FACS Calibur (Beckton Dickinson, San Jose, CA) and CellQuest software.

### *In vivo* studies in transgenic mice

Mice transgenic for the human β-globin gene locus including the locus control region (LCR) in a yeast artificial chromosome (YAC) were previously described with the approval of the Institutional Animal Care and Use Committee of Georgia Regents University[29]. Mice were treated with water as a vehicle control, or Hydroxyurea, 100 mg/kg/dose, administered once daily for 5 days/week, or the lead candidate (Benserazide) administered at 20 mg/kg/dose, (a human equivalent dose of 3 mg/kg), 3 times per week for 5 weeks. Dosing was performed by intraperitoneal injection to ensure consistent drug delivery. Water was administered in the same volume (100 microliters) as the drug candidates as a control. Blood was sampled for complete blood counts and for F-cell quantitation by flow cytometry as previously described[15]. moglobin levels were measured on a Horiba ABX60 at baseline, 2 and 5 weeks (sampling was limited due to constraints on amount of blood that can be withdrawn safely from mice). For flow cytometry analysis, 500,000 cells were washed twice with phosphate buffered, fixed in 4% paraformaldehyde and permeated with ice-cold acetone/methanol (4:1). Cells were incubated with anti-γ-globin-FITC antibody (Santa Cruz Biotechnology, Santa Cruz, CA) in PBT (PBS/01%BSA/0.1% Triton X100) solution for 20 minutes. The FITC-positive cells and mean fluorescence intensity of labeled cells were analyzed by Becton Dickinson LSR-II flow cytometer (BD Bioscience). The test agents were well-tolerated and animals were not sacrificed for the study.

### Statistical Analyses

Data was analyzed by paired t-tests and by a Wilcoxon signed rank test; a level of 0.05 was considered significant.

## Results

### HTS-identified compounds

**Fig 2:**
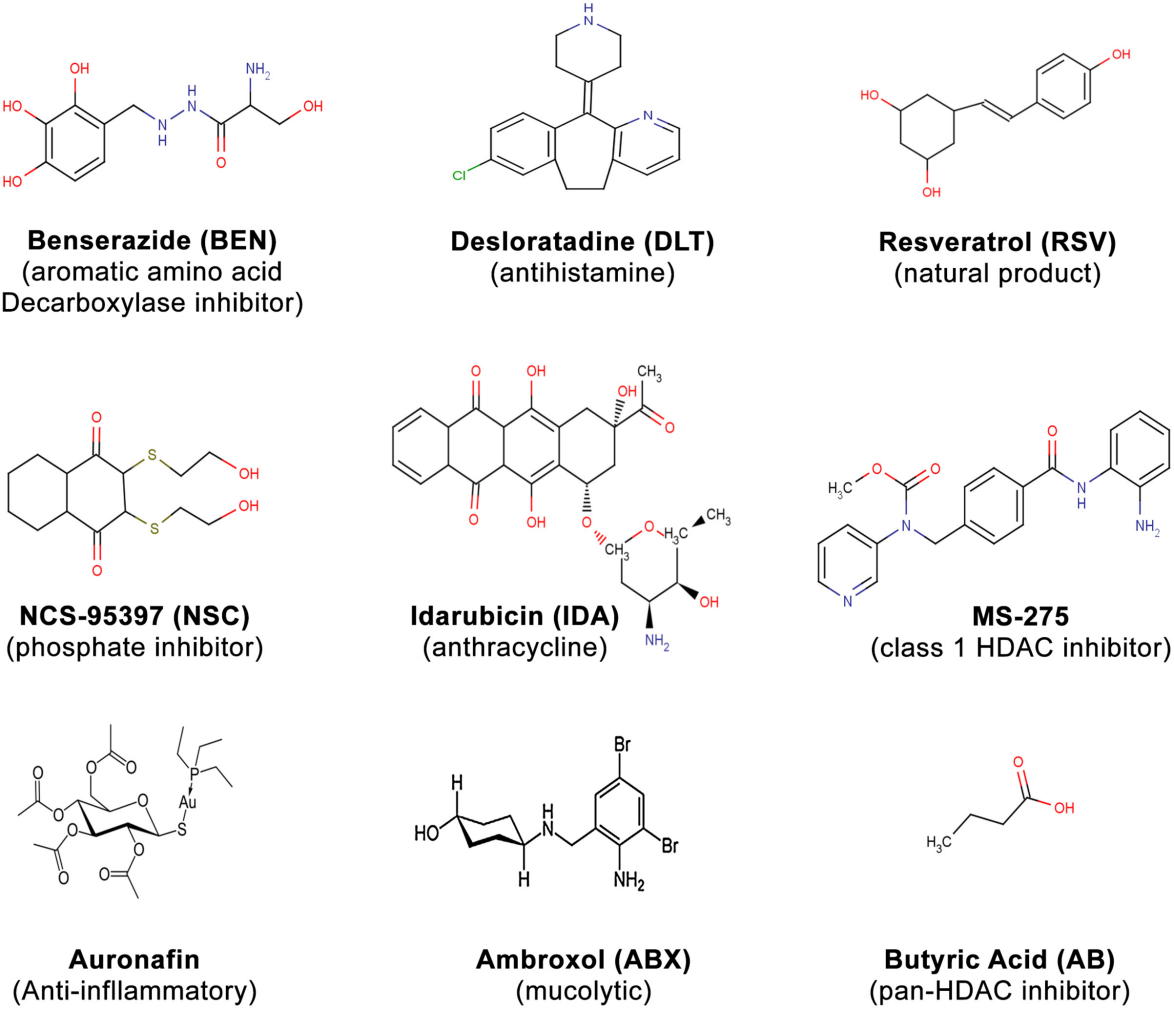
Structures of therapeutic candidates identified in high-throughput screening. Structures of the drug candidates which induce the fetal globin gene promoter are shown. Butyric acid, a known inducer of the fetal globin gene promoter, is shown for comparison.

A schema and read-out example of a positive “hit” are shown in Fig 1. The HTS identified multiple candidates which induced γ-globin gene expression, including some drugs from the EMA- and FDA-approved library (Fig 1), with highly diverse structures. Candidates with oral activity, and/ or benign safety profiles are shown in Fig 2. The structure of a known positive inducer, butyric acid is shown for comparison. High activity was found with Idarubicin, as previously reported[19], which validated the HTS system. The magnitude of induction was similar to the range reported with other high throughput screening campaigns[18]. As only a few candidates did *not* inhibit erythroid cell growth at concentrations that induced γ-globin expression, and/or had routes of administration and safety profiles which appeared suitable for potential therapeutics for hemoglobinopathies, further analyses were focused on those candidates which are orally bioavailable.

### γ-globin induction in erythroid progenitors

**Fig 3:**
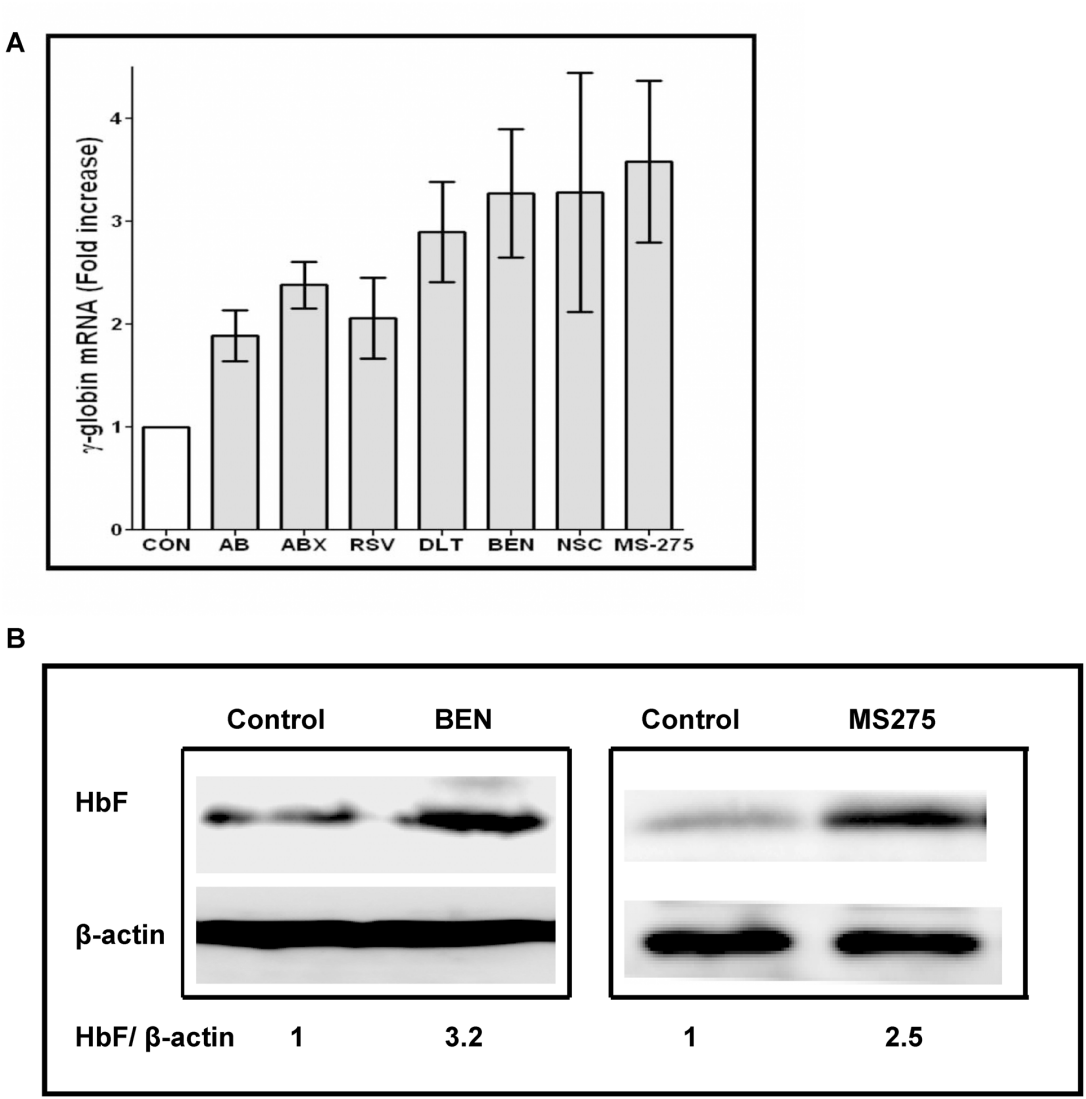
γ-globin mRNA induction in human erythroid progenitors treated with therapeutic candidates. (A.) Mean fold-induction of γ-globin mRNA with addition of Ambroxol (ABX), resveratrol (RSV), Desloratadine (DLT), NSC-95397 (NSC), MS-275, or Benserazide (BEN), compared to vehicle-treated control (Con) in erythroid progenitors from the same subject and to arginine butyrate (AB)-treatment. The range in γ-globin mRNA induction from 2.4- to 4-fold, compared to the subject’s baseline was statistically significant for all candidates and exceeded induction of γ-globin mRNA compared by butyrate (AB) in the same cultures. Error bars indicate standard deviation (SD). (B.) Western blot demonstrating HbF protein induction in erythroid cells treated with therapeutic candidates. HbF protein relative to beta actin is shown below the bands. HbF increased above the ratios in control cells from the same source, by 3.2-fold with Benserazide treatment and by 2.5-fold with MS-275 treatment.

The non-cytotoxic candidates were assayed for their ability to induce γ-globin mRNA in treated erythroid cells compared to untreated progenitors from the same sample. Treatment with the new candidates induced γ-globin mRNA by 2 to 3.9-fold above untreated controls and was significantly different by paired t-tests, p< 0.01, as shown in Fig 3A. A positive control, arginine butyrate, induced by a mean of 1.9-fold, p = 0.01, in the same cells. Expression of HbF at the protein level was confirmed by Western blot; representative examples of Benserazide- and MS-275-treated erythroid cells demonstrated 3.2-fold and 2.5-fold greater HbF, respectively, than in untreated control cells from the same source, shown in Fig 3B.

### γ-globin mRNA induction in non-human primates

**Fig 4:**
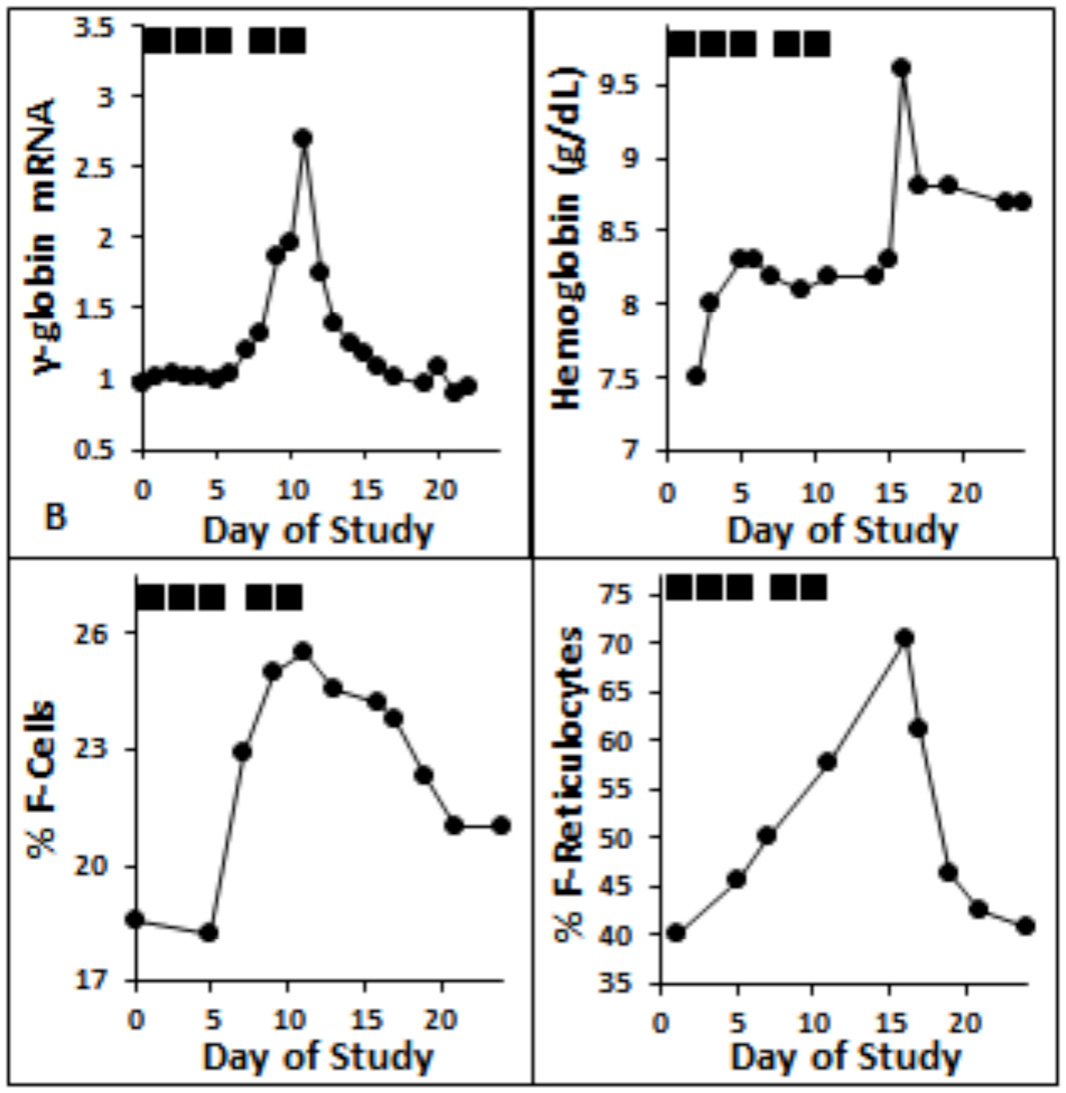
Fetal globin induction in an anemic baboon treated with MS-275 (class I HDAC inhibitor). (A.) Treatment with MS-275 (0.2 mg/kg/dose three times/week), shown by the black bars above the graph, resulted in a 2.7-fold induction of γ-globin mRNA, (B.) 7% increase in F-cells, (C.) 30% increase in F reticulocytes, and (D.) a 1 g/dl rise in total hemoglobin despite daily phlebotomy of 6 ml/kg.

**Fig 5:**
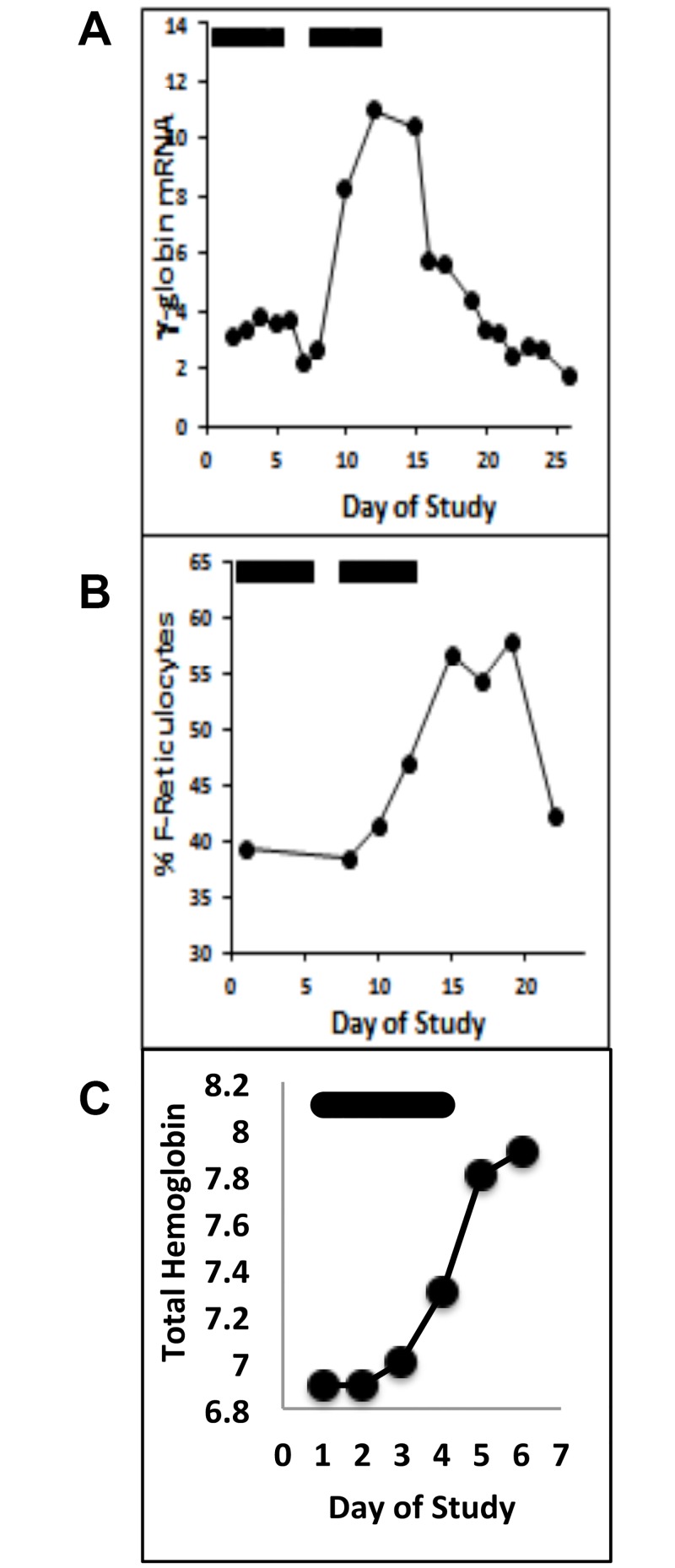
Fetal globin induction in an anemic baboon treated with Desloratidine (DLT). (A.) Treatment with DLT (once per day, 5 days/week), shown by the black bars above the graph, resulted in 5-to 11-fold induction in γ-globin mRNA, (B.) a 15% increase in F-reticulocytes, and (C.) a 1.0 gm/dL increase in total hemoglobin.

**Fig 6:**
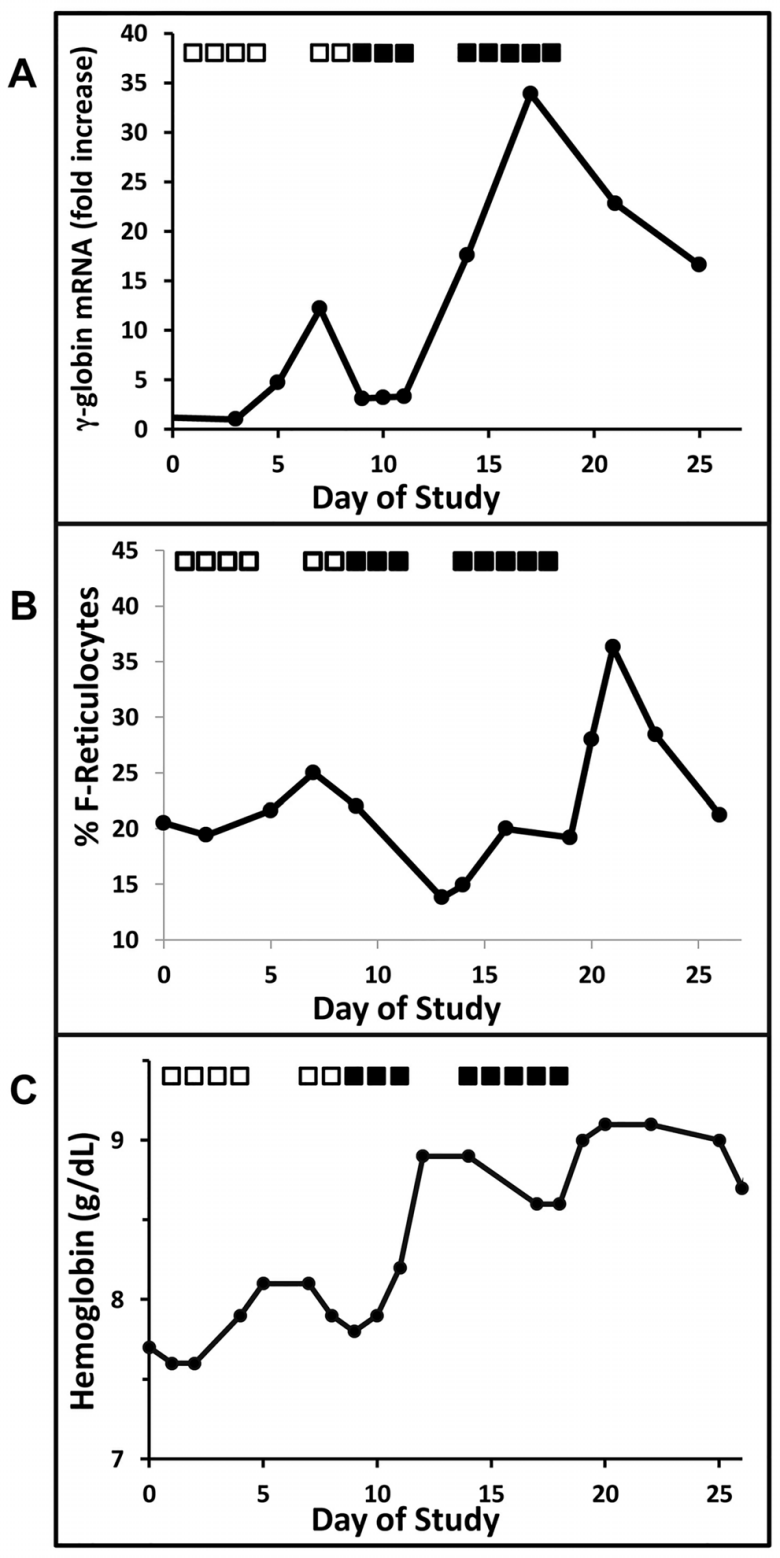
Fetal globin induction in an anemic baboon treated with Benserazide. (A.) Treatment with Benserazide resulted in a dose-dependent increase in γ-globin mRNA up to 34-fold. Two doses were tested, 1 mg/kg/dose (shown by the open squares) and 2 mg/kg/dose, (shown by the dark squares). (B.) F-reticulocytes increased by 15% during treatment with 2 mg/kg, and (C.) total hemoglobin increased by 1.5 gm/dl, despite the phlebotomy which exchanged the blood volume every 12 days.

**Fig 7:**
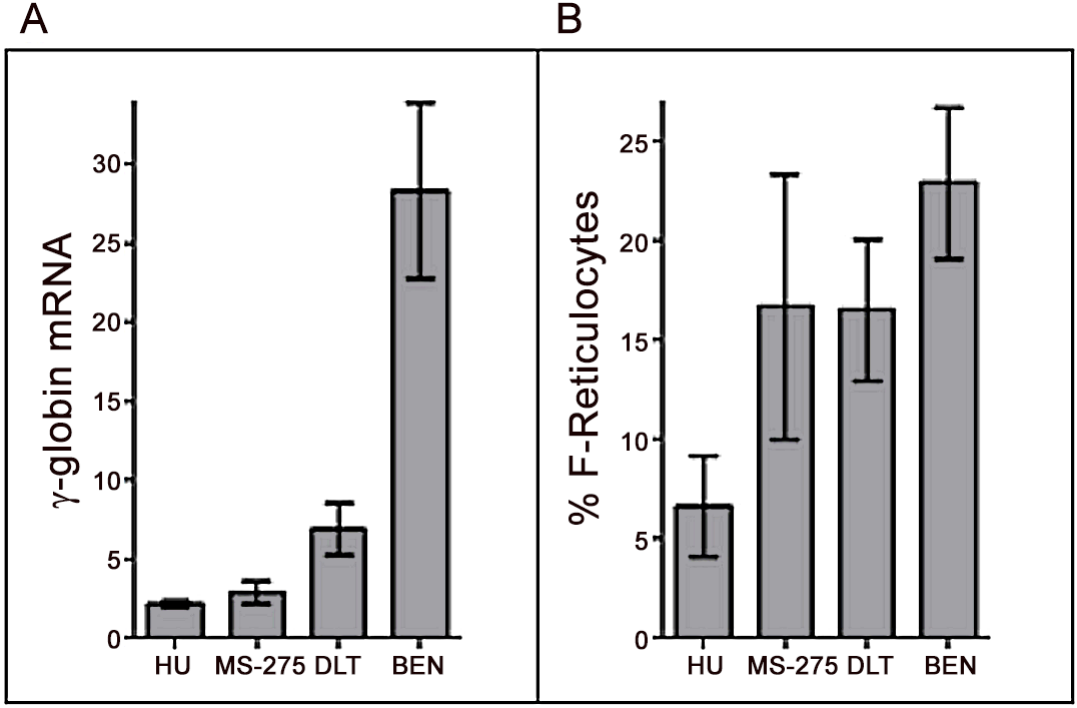
Comparison of fetal globin induction in anemic baboons treated with lead and known candidates. (A.) The magnitude of γ-globin mRNA induction with treatment with Hydroxyurea (HU), MS-275, DLT (mean 7-fold), and Benserazide (27-fold) are shown, and (B.) % F-reticulocytes with the drug treatments are shown. Error bars indicate standard deviation (SD).

Three candidates, Desloratadine (DLT), MS-275 (Etinostat), and Benserazide, which are known to have benign safety profiles with long-term clinical use, were selected for further investigation *in vivo* in anemic baboons. The candidates were compared over 2 weeks of administration following a two-week wash-out period between each drug exposure. Administration of MS-275 (0.2 mg/kg given 3 times/week for two weeks) produced a 2.7-fold peak increase above baseline in γ-globin mRNA, an increase in F-cells from 18% to 26%, and an increase in F-reticulocytes from 40% to 70% (Fig 4). An increase in total hemoglobin from 7.5 to 9.0 gm/dl was observed, despite the ongoing phlebotomy (Fig 4). In an anemic baboon treated with DLT (0.5 mg/kg given three times/week for two weeks), γ-globin mRNA increased by 5.5-fold over baseline in the second week of treatment, and the effect persisted for four days after administration of the last dose, consistent with the time-frame required for erythroid cell differentiation in the baboon (Fig 5). Proportions of F-reticulocytes increased by 19%, from 39% to 58%, (33% of the baseline value), and the increase persisted for 5–10 days following drug administration. Administration of Benserazide (1 mg/kg given once/day orally, 3 times/week for 2 weeks, or 2 mg/kg, 5 days/week) produced the most dramatic increase in γ-globin mRNA. A 12-fold induction in γ-globin mRNA was observed after 1 mg/kg dose, and 27-to 33-fold increase above baseline levels was observed after 2 mg/kg/doses (p<0.01) (Fig 6A). Although changes in HbF levels are difficult to detect with brief 2-week treatment courses, increases in proportions of red blood cells expressing HbF *protein*, F-reticulocytes, increased from 14 to 36% after 2 mg/kg doses (61% of baseline) (Fig 6B). Total hemoglobin increased from 7.6 g/dl at treatment initiation to 9.0 g/dl, despite continued phlebotomy of 4 mls blood/kg/day, which exchanged the baboon’s blood volume approximately every 12 days (Fig 6C). Fig 7 shows a comparison of peak induction of γ-globin mRNA and F-reticulocytes observed in the anemic baboons treated with these agents and compared to effects of Hydroxyurea (HU) or sodium 2,2 dimethylbutyrate (ST20). Small changes in total HbF were detected by HPLC after the brief 2-week treatment courses as follows: with DLT, from 2.5 to 5.6%, MS-275 from 0.6 to 1.3%, and Benserazide from 0.6 to 4.35%.

### Effects of HTS drugs in β-globin locus transgenic mice

**Fig 8:**
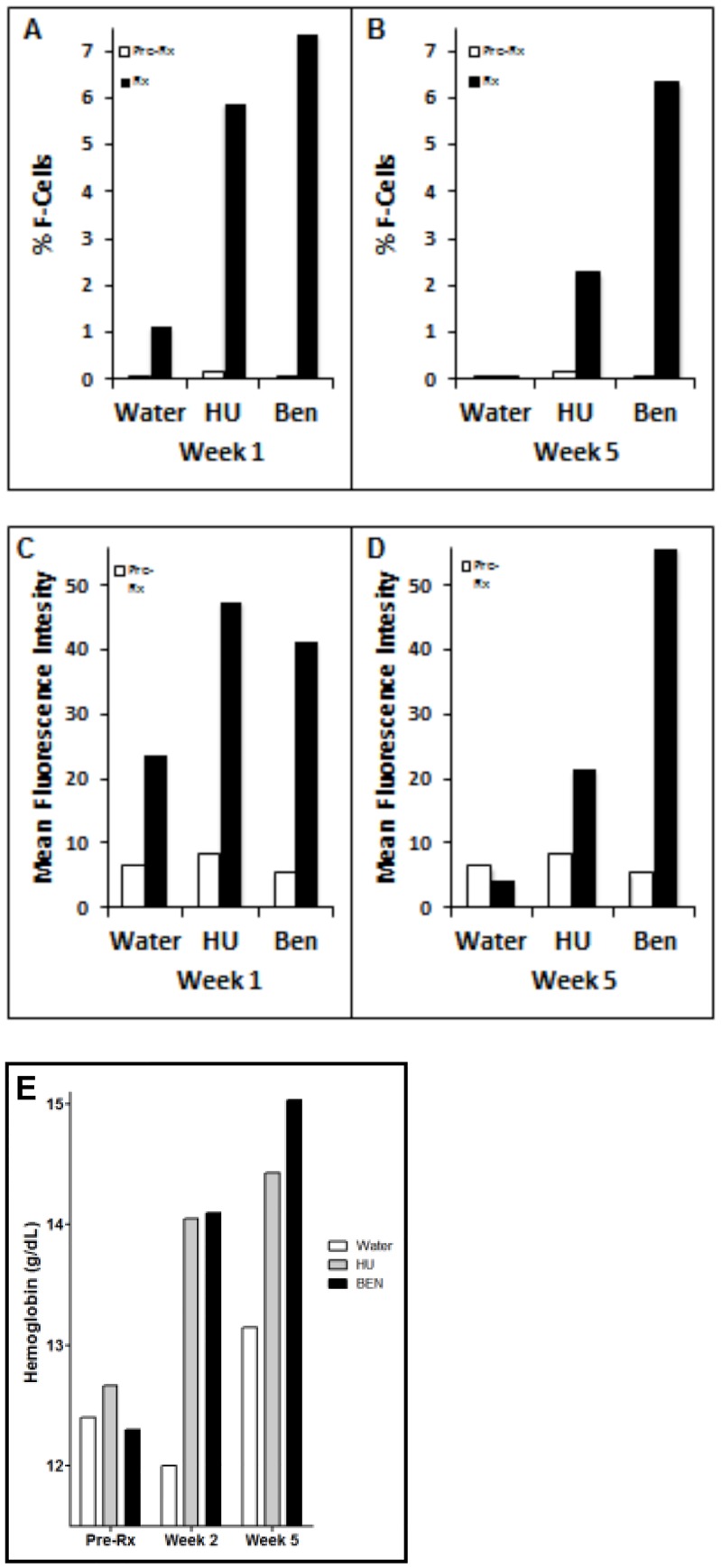
Responses of transgenic mice to treatment with Benserazide or Hydroxyurea. (A. & C.) Mice containing the human non-alpha globin genes in a YAC were treated with vehicle (water) as a control, Hydroxyurea (HU), or Benserazide. % F-cells and mean fluorescent intensity are shown pretreatment *vs*. 2 weeks after initiation of treatment, and (B. & D) pretreatment *vs*. 5 weeks after initiation of treatment. Changes with treatment were significant, (p<0.001 for Benserazide and p< 0.05 for Hydroxyurea). (E.) Total hemoglobin levels are shown pre- treatment *vs*. 2 weeks and 5 weeks after initiation of each treatment.

Mice transgenic for a YAC containing the human LCR-β-globin gene locus have been shown to faithfully recapitulate the human fetal to adult switch, and to respond to prior generation γ-globin inducing agents [29, 35]. Analyses of F-cells in this human β-globin complex transgenic murine model treated with Hydroxyurea or Benserazide at baseline, week 2 (top left panel) and week 5 of treatment (top right panel) are shown in Fig 8A–8D. Mean values in three animals are compared. Proportions of F-cells and mean fluorescence intensity (MFI), both measures of HbF protein, increased significantly within the first week of Hydroxyurea treatment; F-cells increased from 1.2% to 5.7% and MFI increased from 24% to 47% (Fig 8A and 8C); these values declined to 2% F-cells and 13% MFI by week 5, perhaps associated with marrow suppression (Fig 8B and 8D). Treatment with Benserazide (BEN) resulted in an increase in F-cells from 0.7% to 7.3% at week 2, and this level persisted at week 5; MFI increased from 24 to 41% at week 2 and to 50% at week 5 with Benserazide treatment. The changes with the two drug treatments were statistically significant compared to the (water) control values, p<0.0001 with Benserazide treatment and p<0.04 with Hydroxyurea (Wilcoxon signed rank test). Hemoglobin increased from 12 g/dL to 14 g/dL by week 2 with both treatments, and by another 1 to 1.5 gm/dL by week 5 with Benserazide treatment (Fig 8E), suggesting an independent effect on erythropoiesis.

## Discussion

Multiple compounds are reported to induce expression of fetal globin, although only one therapeutic, Hydroxyurea (HU), is currently FDA-approved for treatment of sickle cell disease, and considered ameliorating in approximately 50% of adult patients, causing a mean 3% rise in HbF which reduces morbidity in many treated subjects, and increases survival in those who obtain total HbF of 0.5 g/dl[3,6,8, 11–39]. Additional therapeutics should beneficial for treatment of the diverse global hemoglobinopathy population[4–6]. Developing therapies for orphan conditions requires lengthy trials to identify safe dose limits and doses which produce efficacy, with high costs required for clinical development of new chemical entities. This high-throughput screening effort identified structurally- and functionally-*un*related drug candidates with HbF-inducing activity, although many candidates are not suitable or optimal for long-term treatment. For example, the cytotoxic anticancer drug Idarubicin was identified to have demonstrated high activity, as previously reported[19], which validated the HTS system, but this and several other candidates have side-effect profiles which are undesirable for treatment of a nonmalignant chronic condition. However, a few previously unrecognized small molecules were found to efficiently induce the fetal globin gene promoter, including two candidates approved for treatment of other conditions which have decades of clinical use. Their activity was verified in erythroid cell progenitors and predictive animal models.

Desloratadine, an oral, long-acting tricyclic antihistamine which is approved virtually world-wide, is used to treat symptoms of allergic reactions by blocking the histamine receptor (H1), and preventing activation of H1 receptor-containing cells. Benserazide, an inhibitor of decarboxylation of aromatic amino acids in peripheral (extracerebral) tissues, is used medically to enhance the pharmacokinetic profile of Levodopa, which results in higher concentrations of dopamine in the brain, thus lessening the side-effects observed with higher doses of Levodopa alone[40]. Benserazide is commercially provided in an oral combination tablet with Levodopa for treatment for Parkinson’s disease, and has been approved and widely utilized in this combination for more than 40 years[40] Three other inducers which were not pursued in animal models included: Ambroxol, a drug with secretolytic and secretomotoric actions that restores respiratory physiological clearance mechanisms in bronchopulmonary diseases, which is used as an inhalant. Resveratrol (3,5,4'-trihydroxy-*trans*-stilbene), a naturally-occurring compound with diverse actions, is rapidly metabolized to multiple metabolites in humans, and therefore was not evaluated in these models *in vivo*. The bioactive NSC-95397 showed potent inducing activity in vitro, but blocks G2/M cell cycle phase transition and cell growth, which is undesirable in beta thalassemia, so it was also not evaluated *in vivo*.

The three candidates which have favorable human safety profiles were compared in the anemic baboon model, and their activity was compared to fetal globin induction observed with the established fetal globin inducers Hydroxyurea and sodium 2, 2 dimethylbutyrate (ST20 or HQK-1001)[29–31]. Desloratidine induced γ-globin mRNA up to 11-fold above baseline, MS-275 induced by 2.7-fold, while Benserazide induced by 20- to 33-fold above baseline in two different treated baboons. In beta YAC transgenic mice, F-cells increased within 2 weeks of initiation of Benserazide treatment at 20 mg/kg, compared to HU at 100 mg/kg/dose, and higher proportions of F-cells persisted with Benserazide treatment after 5 weeks than with HU. Human equivalent doses projected from effective doses of Benserazide in the baboon are 0.5 to 1.5 mg/kg/dose for an adult human, which is a lower range than the standard daily doses of 200–300 mg total typically used in an average 70 kg adult with Parkinson’s disease [40]. The benign safety profile of Benserazide, utilized chronically for >40 years[40], with strong fetal globin-inducing activity in baboons and transgenic mice, strongly suggest that clinical evaluation of this therapeutic in patients with hemoglobin disorders is warranted. HbF levels have not been monitored in these patients, to our knowledge.

Many compounds have been found to stimulate γ-globin gene expression, in preclinical systems, acting through a variety of cellular and molecular mechanisms[4–6, 11–13, 17, 18, 37, 38]. The cytotoxic agents, were initially thought to accelerate the maturation of erythroid precursors and produce progeny which maintained the “fetal stage” of globin switching. Other investigators have generated evidence that these drugs may produce a cellular stress response or other signaling pathways that contribute to fetal globin expression[34–37]. Agents which act “directly” on the γ-globin promoter include a variety of compounds of which some disrupt repressor complexes regulating γ-globin gene expression[3–6,11–13,17]. The down-regulation of BCL11A by butyrate[41]. and other short-chain fatty acid derivatives such as sodium 2,2 dimethylbutyrate, may alter the ability of BCL11A to recruit the LCR to specific β-globin complex genes[6,11–13, 17]. HDAC inhibitors, particularly inhibitors of HDACs 1, 2 and 3, block the enzymatic activity of these HDAC isotypes[38] contained in the NURD/CoREST repressor complexes which silence γ-globin expression, releasing this repression and restoring transcription[3–6,38]. Other molecules, including short-chain fatty acid derivatives which lack HDAC activity, cause dissociation of HDAC3 from these same complexes, also releasing transcriptional repression[12–13]. Initial studies of mechanisms of action indicate Benserazide treatment of erythroid progenitors results in displacement of HDAC-3, LSD-1, and suppression of BCL-11A [6] (manuscript in press). Translation of potential therapies in the diverse hemoglobinopathy population is likely affected by underlying molecular mutations, genetic modifier traits[42–50], and by physiologic factors such as erythrokinetics and endogenous erythropoeitin levels[6]. Multiple therapeutics which act through different, potentially complementary mechanisms, may be required to successfully translate this approach in severely affected patients.

The fetal globin inducing activity demonstrated in two animal species here strongly suggests that Benserazide should have activity in human patients, because these animal models have been predictive of activity in subsequent human clinical trials of 5-azacytidine, Butyrate, Isobutyramide, and sodium 2,2 dimethylbutyrate. These earlier generation candidates induced fetal globin expression in baboons generally by 1.5 to 2-fold, and subsequently in clinical trials induced fetal globin protein in patients in thalassemia patients up to 20%. Benserazide emerged as a lead candidate for clinical evaluation due to its efficacy *in vivo* and because it has shown safety with chronic human use for decades in many countries[40]. MS-275 also offers particular interest due to its long half-life, allowing once/week administration, and because histone acetylation, which enhances chromatin accessibility, is likely to facilitate efficacy of other types of γ-globin inducers, offering an approach for combination therapies.

In summary, drug candidate hits from a high-throughput screen employing the human fetal globin gene promoter linked to an EGFP reporter were validated with *in vivo* responses demonstrated in two predictive animal models, including nonhuman primates. Whether select populations of patients with certain mutations or genetic modifiers will respond better to any single agent than others must be evaluated in clinical trials. Previously unrecognized, yet approved, therapeutic candidates discovered in this system offer a development route with lower risk than new chemical entities for which safety profiles and effective administration schedules must be determined.

## References

[1] Weatherall DJ. The inherited diseases of hemoglobin are an emerging global health burden. *Blood*. 2010;115:4331–4336. 10.1182/blood-2010-01-251348 20233970 PMC2881491

[2] Vichinsky EP, MacKlin EA, Waye JS, Lorey F, Olivieri NF. Changes in the epidemiology of thalassemia in North America: a new minority disease. *Pediatrics*. 2005; Dec;116(6):e818–25. 16291734 10.1542/peds.2005-0843

[3] Steinberg MH, Rodgers GP. Pharmacologic modulation of fetal hemoglobin.Medicine. 2001;80: 328–344. 11552087 10.1097/00005792-200109000-00007

[4] Wilbur A, Neinhuis AW, Person DA. Transcriptional regulation of fetal to adulthemoglobin switching: new therapeutic opportunities. *Blood* 117:3945–3953, 2011 10.1182/blood-2010-11-316893 21321359 PMC3087525

[5] Bauer DE, Kamran SC, Orkin SH. Reawakening fetal hemoglobin: prospects for new therapies for the beta- globin disorders. *Blood*. 2012;120(15):2945–2953. 10.1182/blood-2012-06-292078 22904296 PMC4467860

[6] Perrine SP, Pace BS, Faller DV. Targeted fetal hemoglobin induction for treatment of beta hemoglobinopathies In: Vichinsky EP, editor. Emerging therapy in hemoglobinopathies: lessons from the past and optimism for the future. Hematol Oncol Clin North Am. pp 233–48.ed. Philadelphia, PA: Elsevier, Inc.; 2014 10.1016/j.hoc.2013.11.009 24589264

[7] Platt OS, Brambilla DJ, Rosse WF, Milner PF, Castro O, Steinberg MH, et al Mortalityin sickle cell disease. Life expectancy and risk factors for early death. *N Engl J Med*. 1994; 330:1639–1644. 7993409 10.1056/NEJM199406093302303

[8] Steinberg MH, McCarthy WF, Castro O, Ballas SK, Armstrong FD, Smith W, et al Therisks and benefits of long-term use of hydroxyurea in sickle cell anemia: A 17.5 year follow-up. *Am J Hematol*. 2010;85:403–408. 10.1002/ajh.21699 20513116 PMC2879711

[9] Schrier SL. Pathobiology of thalassemic erythrocytes. *Curr Opin Hematol*. 1997;4:75–78. 9107522 10.1097/00062752-199704020-00001

[10] Taher AT, Musallam KM, Karimi M, El-Beshlawy A, Blhoul K, Daar S, et al Overviewon practices in thalassemia intermedia management aiming for lowering complicationsrates across a region of endemicity: the Optimal Care Study. Blood. 2010; 11:1886–1892. 10.1182/blood-2009-09-243154 20032507

[11] Bohacek R, Boosalis MS, McMartin C, Faller DV, Perrine SP. Identification of novel small-molecule inducers of fetal hemoglobin using pharmacophore and 'PSEUDO' receptor models. *Chem Biol Drug Des*. 2006;67:318–328. 16784456 10.1111/j.1747-0285.2006.00386.x PMC4263278

[12] Perrine SP, Mankidy R, Boosalis MS, Bieker JJ, Faller DV. Erythroid Kruppel-like factor (EKLF) is recruited to the gamma-globin gene promoter as a co-activatorand is required for gamma-globin gene induction by short-chain fatty acid derivatives. *Eur J Haematol*. 2009; 82:466–476. 10.1111/j.1600-0609.2009.01234.x 19220418 PMC3232177

[13] Mankidy R, Faller DV, Mabaera R, Lowrey CH, Boosalis MH, White GL, et al Short-chain fatty acids induce gamma-globin gene expression by displacement of aHDAC3-NCoR repressor complex. *Blood*. 2006;108:3179–3186. 16849648 10.1182/blood-2005-12-010934 PMC1895523

[14] Constantoulakis P, Knitter G, Stamatoyannopoulos G. On the induction of fetal hemoglobin by butyrates: in vivo and in vitro studies with sodium butyrate and comparison of combination treatments with 5-AzaC and AraC. Blood. 1989;74:1963–1971. 2478217

[15] Pace BS, White GL, Dover GJ, Boosalis MS, Faller DV, Perrine SP. Short-chain fatty acid derivatives induce fetal globin expression and erythropoiesis in vivo. *Blood*. 2002; 100:4640–4648. 12393583 10.1182/blood-2002-02-0353 PMC4269367

[16] Boosalis MS, Bandyopadhyay R, Bresnick EH, Pace BS, Van Demark K, Zhang B, et al Short-chain fatty acid derivatives stimulate cell proliferation and induce STAT-5 activation. *Blood*. 2001;97:3259–3267. 11342457 10.1182/blood.v97.10.3259 PMC4263369

[17] Cui S, Lim K-C, Shi L, Lee M, Jearawiriyapaisarn N, Myers G, et al The LSD1 inhibitor RN-1 induces fetal hemoglobin synthesis and reduces disease pathology in sickle cell disease.Blood. 2015; 10.1182/blood-2015-02-626259 PMC4504950 26031919

[18] Peterson KR, Costa FC, Fedosyuk H, Neades RY, Chazelle AM, Zelenchuk L, et al A cell-based high-throughput screen for novel chemical inducers of fetal hemoglobin for treatment of hemoglobinopathies.PloS One. 2014;9 16;9(9):e107006 eCollection 2014. 25225870 10.1371/journal.pone.0107006 PMC4165891

[19] Spyrou P, Phylactides M, Lederer CW, Kithreotis L, Kirri A, Christou S, et al Compounds of the anthracycline family of antibiotics elevate human γ-globin expression both in erythroid cultures and in a transgenic mouse model. *Blood Cells Mol Dis*. 2010;44:100–106. 10.1016/j.bcmd.2009.10.008 19914848

[20] DeSimone J, Heller P, Hall L, Zwiers D. 5-azacytidine stimulates fetal hemoglobin synthesis in anemic baboons. *Proc Natl Acad Sci USA*. 1982;79(14):4428–4431 6181507 10.1073/pnas.79.14.4428 PMC346685

[21] Perrine SP, Ginder GD, Faller DV, Dover GH, Ikuta T, Witkowska HE, et al A short-term trial of butyrate to stimulate fetal-globin-gene expression in the beta-globin disorders. *N Engl J Med*. 1993;328:81–86. 7677966 10.1056/NEJM199301143280202

[22] Collins AF, Pearson HA, Giardina P, McDonagh KT, Brusilow SW, Dover GJ. Oral sodium phenylbutyrate therapy in homozygous beta thalassemia: a clinical trial. *Blood*. 1995; 85:43–49. 7528572

[23] Atweh GF, Sutton M, Nassif I, Boosalis V, Dover GJ, Wallenstein S, et al Sustainedinduction of fetal hemoglobin by pulse butyrate therapy in sickle cell disease. *Blood*. 1999;93:1790–1797. 10068649 PMC4269326

[24] Resar LM, Segal JB, Fitzpatric LK, Friedmann A, Brusilow SW, Dover GJ. Induction of fetal hemoglobin synthesis in children with sickle cell anemia on low-dose oral sodium phenylbutyrate therapy. *J Pediatr Hematol Oncol*. 2002;24:737–741. 12468915 10.1097/00043426-200212000-00011

[25] Perrine SP, Dover GH, Daftari P, Walsh CT, Jin Y, Mays A, Faller DV. Isobutyramide, an orally bioavailable butyrate analogue, stimulates fetal globin gene expression in vitroand in vivo. *Br J Haematol*. 1994;88:555–561. 7529533 10.1111/j.1365-2141.1994.tb05073.x

[26] Charache S, Terrin ML, Moore RD, Dover GJ, Barton FB, Eckert SV, et al Effect of hydroxyurea on frequency of painful crises in sickle cell anemia. Investigators of the multicenter study of hydroxyurea in sickle cell anemia. *N Engl J Med*. 1995;332(2):1317–1322. 7715639 10.1056/NEJM199505183322001

[27] Wang WC, Ware RE, Miller ST, Iyer RV, Casella JF, Minniti CP, et al Hydroxycarbamide in very young children with sickle-cell anaemia: a multicentre, randomized, controlled trial (BABY HUG). *Lancet*. 2011;377:1663–1672. 10.1016/S0140-6736(11)60355-3 21571150 PMC3133619

[28] Saunthararajah Y, Hillery CA, Lavelle D, Molokie R, Dorn L, Bressler L, et al Effects of 5- aza-2'-deoxycytidine on fetal hemoglobin levels, red cell adhesion, and hematopoietic differentiation in patients with sickle cell disease. *Blood*. 2003;102:3865–3870. 12907443 10.1182/blood-2003-05-1738

[29] Fuchareon S, Inati A, Siritanaraku N, Thein SL, Wargin WC, Koussa S, et al A randomized Phase I/II trial of HQK-1001, an oral foetal globin gene inducer, in beta thalassemia intermedia and HbE beta thalassemia. *Br J Haematol*. 2013; 5; 161(4): 587–93. 10.1111/bjh.12304 23530969 PMC3970579

[30] Kutlar A, Reid ME, Ataga K, Inati A, Taher AT, Abboud MR, et al A dose escalation phase IIa study of 2, 2-dimethylbutyrate (HQK-1001), an oral fetal globin inducer, in sickle celldisease. *Am J Hematol*. 2013;(11):E255–260. 10.1002/ajh.23533 23828223

[31] Patthamalai P, Fuchareon S, Chaneiam N, Ghalie R, Chui DHK, Boosalis MS, et al Aphase 2 trial in HQK-1001 in HBE-β thalassemia demonstrates HBF induction and reduced anemia. *Blood*. 2014;123(12):1956–7. 10.1182/blood-2013-11-538470 24652964 PMC3962168

[32] Cappelini MD, Graziadei G, Ciceri L, Comino A, Bianchi P, Porcella A, et al Oral isobutyramide therapy in patients with thalassemia intermedia: results of a phase II open study. Blood Cells, Mols, Dis. 2000;26:105–111. 10.1006/bcmd.2000.0283 10772882

[33] Reich S, Buhrer C, Henze G, Ohlendorf D, Mesche M, Sinha P, et al Oral isobutyramidereduces transfusion requirements in some patients with homozygous beta thalassemia. *Blood*. 2000;96:3357–3363. 11071627

[34] Dai Y, Ngo D, Forman LW, Qin DC, Jacob J, Faller DV. Sirtuin 1 is required for an antagonist induced transcriptional repression of androgen responsive genes by the androgen receptor. Mol Endocrinol 2007;21:807–1811. 10.1210/me.2006-0467 PMC3839341 17505061

[35] Mabaera R, West RJ, Conine SJ, Macari ER, Boyd CD, Engman CA, Lowrey CH. A cell stress signaling model of fetal hemoglobin induction: what doesn't kill red blood cells may make them stronger. *Exp Hematol*. 2008;36:1057–1072. 10.1016/j.exphem.2008.06.014 18718415

[36] Chen J-J. Regulation of protein synthesis by the heme-regulated eIF2α kinase: relevance to anemias. Blood. 2007; 109:2693–2699. 17110456 10.1182/blood-2006-08-041830 PMC1852217

[37] Schaeffer EK, West RJ, Conine SJ, Lowrey CH. Multiple physical stresses induce γ-globin gene expression and fetal hemoglobin production in erythroid cells. *Blood Cells Mol Dis*. 2014;52;214–221. 10.1016/j.bcmd.2013.10.007 24314748

[38] Bradner JE, Mak R, Tanquturi SK, Mazitschek R, Haggarty SJ, Ross, et al Chemical genetic strategy identifies histone deacetylase 1 (HDAC1) and HDAC2 as therapeutic targets in sickle cell disease. *Proc Natl Acad Sci USA*. 2010;107(28):12617–12622. 10.1073/pnas.1006774107 20616024 PMC2906581

[39] Testa U. Fetal hemoglobin chemical inducers for treatment of hemoglobinopathies. *Ann Hematol*. 2009;88:505–528. 10.1007/s00277-008-0637-y 19011856

[40] Hoffman-La Roche Limited. Product Monograph levodopa and benserazide combination Capsules 50–12.5, 100–25, 200–50 Pharmaceutical standard: professed Antiparkinson Agent. Submission control No. 128706. Available: http://www.rochecanada.com/fmfiles/re7234008/Research/ClinicalTrialsForms/Products/ConsumerInformation/MonographsandPublicAdvisories/ProlopaPME.pdf. Accessed 2014 August 22.

[41] Chen Z, Luo HY, Steinberg MH, et al BCL11A represses HBG transcription in K562 cells. *Blood Cells Mol Dis*. 2009;42:144–149.2. 10.1016/j.bcmd.2008.12.003 19153051

[42] Labie D, Pagnier J, Lapoumeroulie C, Rouabhi F, Dunda-Belkhodja O, Chardin P, et al Common haplotype dependency of high G gamma-globin gene expression and high Hb F levels in beta-thalassemia and sickle cell anemia patients. *Proc Natl Acad Sci USA*. 1985;82: 2111–2114. 2580306 10.1073/pnas.82.7.2111 PMC397502

[43] Thein SL, Menzel S. Discovering the genetics underlying foetal haemoglobin production in adults. *Br J Haematol*. 2009;145:455–467. 10.1111/j.1365-2141.2009.07650.x 19344402

[44] Sheehan VA, Luo Z, Flanagan JM, Howard TA, Thompson BW, Wang WC, et al Genetic modifiers of sickle cell anemia in the Baby HUG cohort: influence on laboratory and clinical phenotypes. *Am J Hematol*. 2013 4 20; 10.1002/ajh.23457 23606168

[45] Uda M, Galanello R, Sanna S, Lettre G, Sankaran VG, Chen W, et al Genome-wideassociation study shows BCL11A associated with persistent fetal hemoglobin and amelioration of the phenotype of beta-thalassemia. *Proc Natl Acad Sci USA*. 2008;105:1620–1625. 10.1073/pnas.0711566105 18245381 PMC2234194

[46] Nuinoon M, Makarasara W, Mushiroda T, Setianinqsih I, Wahidiyat PA, Sripichai O, et al A genome-wide association identified the common genetic variants influence disease severity in beta 0-thalassemia/hemoglobin *E Hum Genet*. 2010;127:303–314. 10.1007/s00439-009-0770-2 20183929

[47] Lettre G, Sankaran VG, Bezerra MA, Araujo AS, Uda M, Sanna S, Cao A, et al DNA polymorphisms at the BCL11A, HBS1L-MYB, and beta- globin loci associate with fetal hemoglobin levels and pain crises in sickle cell disease. *Proc Natl Acad Sci USA*.2008;105(33):11869–11874. 10.1073/pnas.0804799105 18667698 PMC2491485

[48] Liu D, Zhang X, Yu L, Cai R, Ma X, Zheng C, et al KLF1 mutations are relatively more common in a thalassemia endemic region and ameliorate the severity of β-thalassemia. *Blood*. 2014;124:803–811. 10.1182/blood-2014-03-561779 24829204 PMC4118488

[49] Manwani D, Bieker JJ. KLF1: when less is more. *Blood*. 2014;124:672–673. 10.1182/blood-2014-05-576967 25082863 PMC4118480

[50] Bank A. Regulation of human fetal hemoglobin: new players, new complexities. *Blood*. 2006;107:435–443. 16109777 10.1182/blood-2005-05-2113 PMC1895603

